# Primary testicular natural killer/T-cell lymphoma: A CARE-case report and review of literature

**DOI:** 10.1097/MD.0000000000010181

**Published:** 2018-03-23

**Authors:** Wan-Lin Zhang, Shuang Ma, Rachel Jug, Fan Li, Endi Wang, Huan-Yu Zhao, Hong-Tao Xu, Lin Cai, Cheng-Qian Yu, Shuai Shen, Lian-He Yang

**Affiliations:** a Department of Pathology, First Affiliated Hospital and College of Basic Medical Sciences, China Medical University; b Department of Neurology, Sheng Jing Hospital of China Medical University, Shenyang, Liaoning, China; c Department of Pathology, Duke University Medical Center, Durham, NC, USA; d Department of Library Science, School of Medical informatics, China Medical University, Shenyang, Liaoning, China.

**Keywords:** diagnosis, NK/T-cell lymphoma, pathology, prognosis, testicular

## Abstract

**Rationale:**

Primary testicular natural killer (NK)/T-cell lymphoma is an extremely rare and highly aggressive lymphoid malignancy. At present, only 20 cases have been reported.

**Patient concerns:**

A 32-year-old Chinese man complained of discomfort and swelling of his right testicle for 3 months. Physical examination revealed a 10 × 10 × 9.5 cm mass on the right side of the scrotum area.

**Diagnoses:**

Pathologic evaluation showed effacement of normal testicular parenchymal architecture by small-to-medium-sized lymphoid cells with irregular nuclear profiles, and immunohistochemical studies positively expressed CD2, CD56, cytoplasmic CD3, granzyme B, perforin, and TIA-1. Therefore, the patient was diagnosed with primary testicular NK/T-cell lymphoma.

**Interventions:**

The patient underwent CHOP (cyclophosphamide (CTX), pirarubicin (THP-ADM), vincristine (VCR), and prednisolone (PDN)) chemotherapy.

**Outcomes:**

The patient relapsed 5 months after his initial presentation and died after an infection and gastrointestinal bleed.

**Lessons:**

Clinicopathological assessment of this rare case highlights the clinical and pathological features required to diagnose testicular NK/T-cell lymphoma. In addition, it highlights the dismal survival of these patients. We hope it may serve as a reference aiding prompt clinical diagnosis, which can hopefully improve the survival and quality of life of these patients.

## 1 Introduction

Primary testicular lymphoma (PTL) is an uncommon disease accounting for 1% of non-Hodgkin's lymphoma.^[1]^ The most common histological type of primary testicular lymphoma is diffuse large B-cell lymphoma.^[2]^ Primary testicular NK/T-cell lymphoma is an extremely rare and highly aggressive malignancy. To the best of our knowledge, only 14 cases have been reported in the English literature to date.^[1–11]^ According to the 2016 World Health Organization (WHO) reclassification of hematological malignancies,^[12]^ natural killer cell tumors can be divided into 3 categories, including extranodal NK/T-cell lymphoma (ENKTCL-N), aggressive NK-cell leukemia, and chronic lymphoproliferative disorder of NK cells. There are 2 types of NKTCL-N, nasal type and non-nasal type. The former occurs mainly in the midline structures, including the nasal cavity, nasopharynx, and paranasal sinuses, whereas the latter affects the skin, gastrointestinal tract, soft tissue, spleen, lungs, and testis. Patients with primary testicular NK/T-cell lymphoma usually present with painless testicular enlargement, and often manifest with early dissemination and rapid clinical progression. The disease tends to relapse promptly after initial management involving orchiectomy and chemoradiotherapy, reflective of its poor prognosis. ^[3]^

Herein we report a case of primary testicular NK/T-cell lymphoma in a 32-year-old Chinese man and present our findings from a review of the literature to summarize the key points regarding the diagnosis, prognosis, and clinical treatment of this entity.

## 2 Case presentation

A 32-year-old Chinese man presented with a 3-month history of enlargement and discomfort of his right testicle. The patient was treated with antimicrobial therapy since an infectious etiology was suspected as the cause of his scrotal swelling; however, his symptoms did not resolve. The patient had no family history of malignancy. However, serum tumor markers included alpha-fetoprotein (AFP) 11.97 ng/mL (0.00–7.00 ng/mL), carcinoembryonic antigen (CEA) 2.33 ng/mL (0.00–4.30 ng/mL), glycogen antigen 12-5 (CA12-5) 8.43U/mL (0.00–35.00 U/mL), CA15-3 8.60 U/mL (0.00–25.00 U/mL), CA19-9 13.00 U/mL (0.00–27.00 U/mL), and HCG <0.10 mIU/mL (0.00–3.00 mIU/mL).

**Figure 1:**
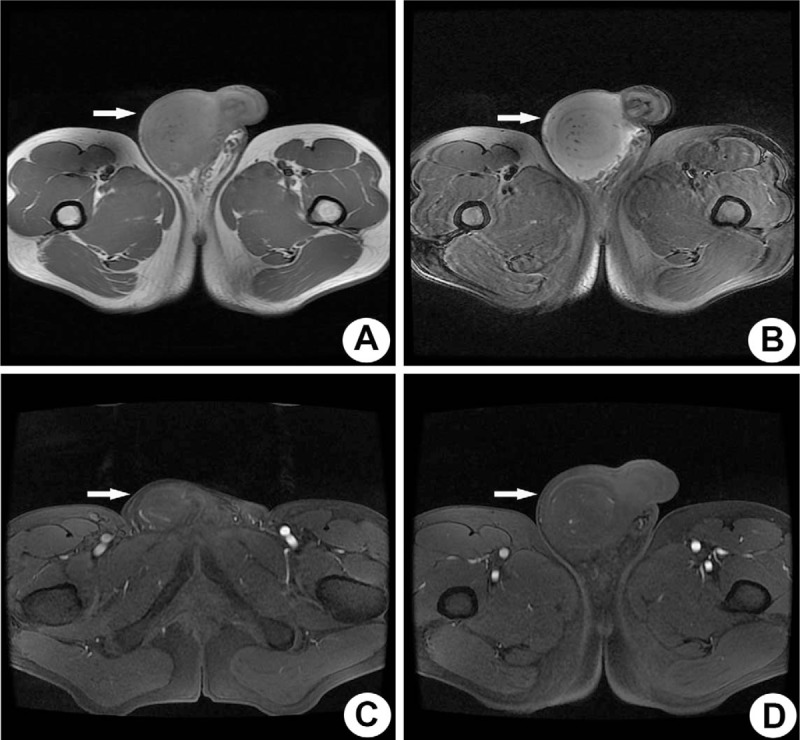
MRI images of primary testicular NK/T-cell lymphoma. (A) and (B) show a significantly enlarged right testicle due to a testicular mass, exhibiting an equal T1 and a slightly longer T2 signal, respectively. (C) and (D) show slightly inhomogeneous enhancement lines and dots in the mass. The lesion was highlighted with a white arrow.

Physical examination revealed a 10 × 10 × 9.5 cm mass on the right side of the scrotal area. Subsequently, a magnetic resonance imaging (MRI) scan showed a significantly enlarged right testis measuring 12.3 × 8.4 × 8.4 cm in size, in which a 6.8 × 6.2 × 6.5 cm mass/shadow could be seen. The mass exhibited equal T1 signal (Fig. 1A) and a slightly longer T2 signal (Fig. 1B), with accompanying faintly inhomogeneous enhancements consisting of lines and dots (Fig. 1C and D). A right radical orchiectomy was preformed and the testicle was submitted for pathological examination. Lymph node metastases and distant metastases were not identified. Grossly, the 15 × 15 × 12 cm-sized mass nearly occupied the entire testis, invaded the tunica vaginalis of testis, and adjacent tissue. The cut surface of the mass was white to gray in color and firm in texture.

**Figure 2:**
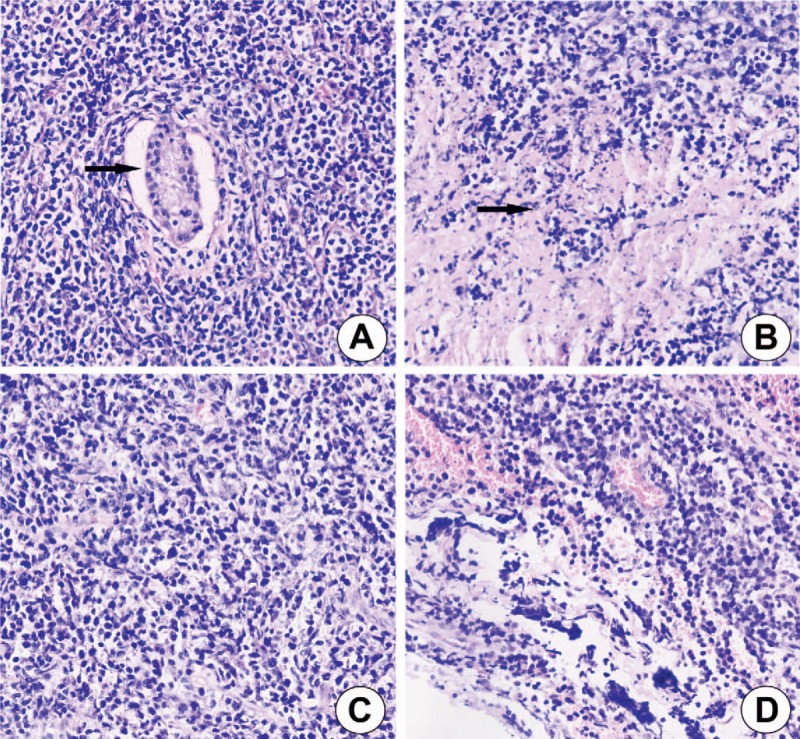
Histopathological features of primary testicular NK/T-cell lymphoma. (A) Numerous tumor cells effaced the normal testicular tissue with only few residual atrophic seminiferous tubules identified (H&E, 200 × magnification). (B) Focal necrosis could be found easily (H&E, 200 × magnification). (C) Most tumor cells have irregular nuclei, scant cytoplasm, lack prominent nucleoli, with a small subset containing nucleoli and clear cytoplasm with a signet-ring cell appearance (H&E, 200 × magnification). (D) Tunica vaginalis involved by tumor (H&E, 200 × magnification).

**Figure 3:**
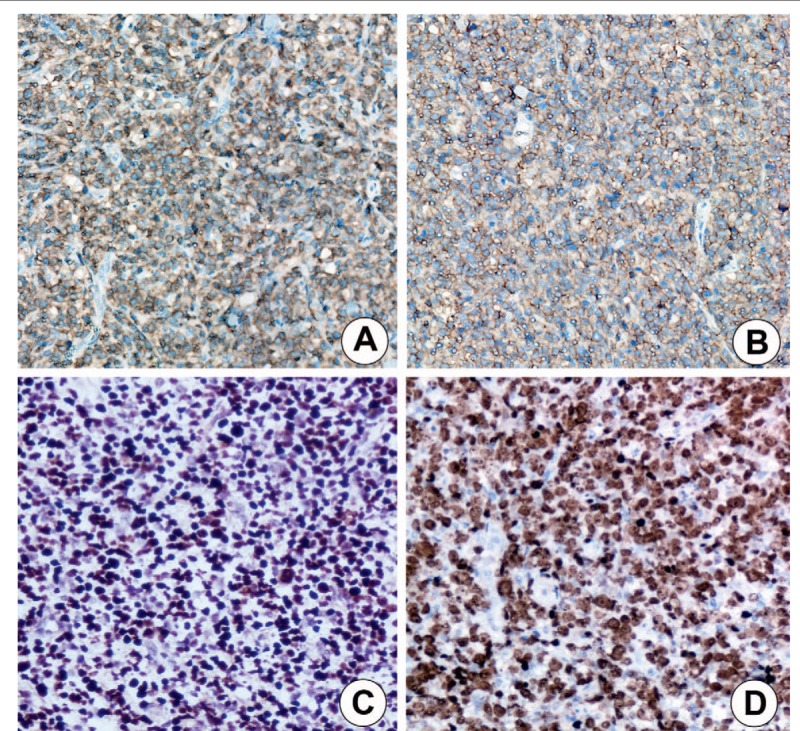
Immunohistochemical staining pattern of primary testicular NK/T-cell lymphoma. (A) Strong positive cytoplasmic reaction for CD3 (IHC staining, 200 × magnification). (B) Strong positive membranous staining for CD56 (IHC staining, 200 × magnification). (C) In situ hybridization showed nuclear positivity for Epstein–Barr virus-encoding RNA (200 × magnification). (D) The hottest area of Ki67 approached 90%, consistent with a strong positive result (IHC staining, 200 × magnification).

Histologically, the normal testicular architecture was effaced and replaced by seminiferous tubules (Fig. 2A) and necrosis (Fig. 2B). The tumor cells were small to medium sized with irregular nuclear contours, nuclear pleomorphism, minimal cytoplasm, and lacked prominent nucleoli. Occasional tumor cells had clear cytoplasm with a signet-ring cell appearance. A subset exhibited oval-shaped nuclei and small nucleoli (Fig. 2C). Numerous tumor cells infiltrated the tunica vaginalis (Fig. 2D). Immunohistochemical (IHC) studies demonstrated tumor cells staining positively with CD2, granzyme B, perforin, TIA-1, cytoplasmic CD3 (Fig. 3A), and membranous CD56 (Fig. 3B). In situ hybridization showed tumor cell positivity for Epstein–Barr virus-encoding RNA (EBER) (Fig. 3C). In addition, the high proliferation index demonstrated by Ki-67 immunohistochemical staining focally approached 90% (Fig. 3D). Tumor cells displayed negative immunoreactivity for CD4, CD5, CD7, CD8, CD20, CD30, and PAX-5. This lesion was pathologically confirmed as primary testicular NK/T-cell lymphoma. The mass was pathologically confirmed as NK/T-cell lymphoma. Unfortunately, the patient discontinued CHOP chemotherapy (day 1: CXT 750 mg/m^2^, THP-ADM 50 mg/m^2^, VCR 1.4 mg/m^2^, PND 100 mg/m^2^; days 2–7: PND 100 mg/m^2^; day 8: CXT 750 mg/m^2^, PND 100 mg/m^2^; q21d; 8 cycles.) after 3 treatment cycles due to side effects experienced. He died secondary to infection and a gastrointestinal bleed 5 months later.

## 3 Discussion

Primary testicular NK/T-cell lymphoma is an extremely rare and aggressive malignant neoplasm characterized by morphological features resembling PTL, with aberrant CD56 and EBV expression. Table 1 summarizes clinical and immunohistochemical features of NK/T-cell lymphoma from the previous published reports between 1993 and 2016. To the best of our knowledge, only 20 cases of primary testicular NK/T-cell lymphoma have been reported with 14 cases in the English-language literature and 6 cases in the Chinese-language literature. This condition has a relatively higher incidence in Asia, especially in southern China. The age of patients included in the published cases ranges from 28 to 71 years with a median age of 49.5 years. The majority of lesions present unilaterally with accompanying significant testicular enlargement. There is no standard therapy or known effective treatment for primary testicular NK/T-cell lymphoma at present^[4]^; therefore, orchiectomy is the mainstay of treatment, whereas radiotherapy and chemotherapy (mainly CHOP—cyclophosphamide, doxorubicin, vincristine, and prednisolone) have been utilized as adjuvant treatments with no curative success. Unfortunately, the efficacy of treatments published has been dismal. A review of the literature revealed except for 5 cases lost follow-up, all patients relapsed promptly and died of complications of their disease and treatment of such, including gastrointestinal bleeding, peritonitis, and acute multiorgan failure.^[8,13–15]^ Given these observations, we firmly believe that NK/T-cell lymphoma involving the testis should be recognized as a distinct and highly aggressive entity.

**Table 1:**
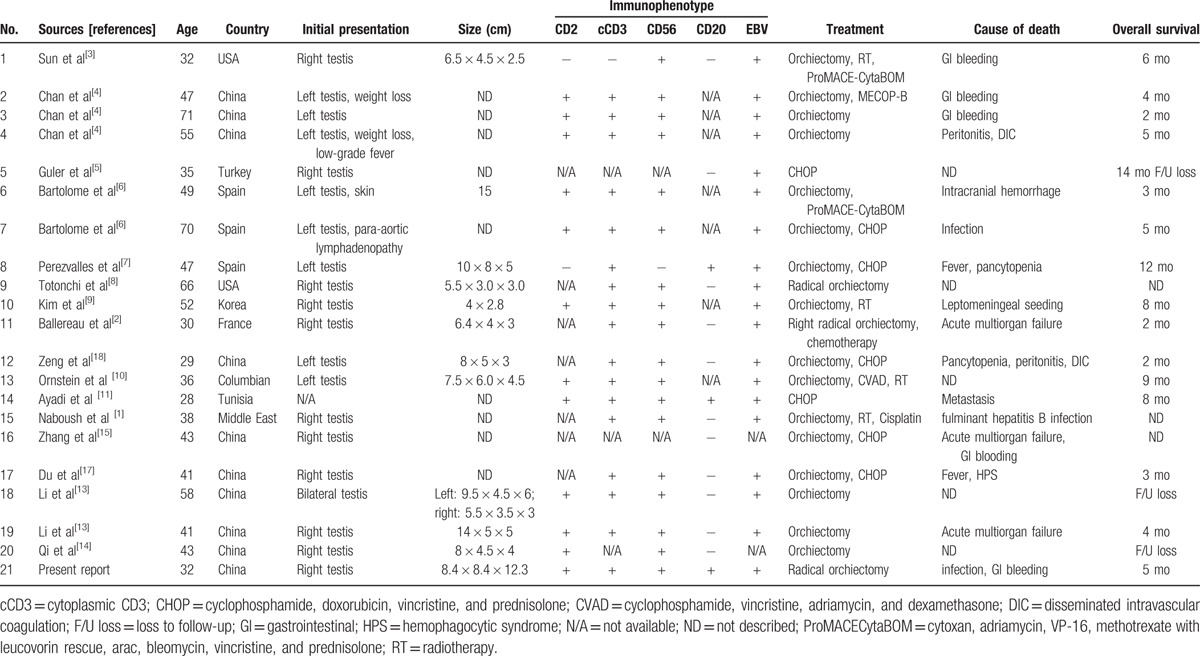
Clinical features of testicular NK/T-cell lymphoma.

The surface glycoprotein CD56 is a neuronal cellular adhesion molecule (NCAM) that possesses homophilic binding properties and has been found in a variety of normal tissues such as brain, nerve, muscle, and natural killer cells.^[5]^ In additional, it is expressed by other neoplasms.^[16]^ Guler et al found that the sites richly expressing CD56 possess homing properties and combine with CD56-positive lymphoma cells, such as can be seen in the upper aerodigestive tract, skin, soft tissue, gastrointestinal (GI) tract, spleen, and testes.^[5]^ The fact that NK/T-cell lymphoma has been seen to spread to such sites in such a short time span may support this homing phenomenon and provide an explanation for its capability for widespread dissemination. According to previously reported cases, almost all patients rapidly died of various complications <6 months after diagnosis, regardless of modern treatment modalities administered.^[1–9,11,13,14,17,18]^

It is worth noting that EBV genomes were detected in all reported cases of primary testicular NK/T-cell lymphoma. In situ hybridization or PCR revealed the positive expression of the EBV in the previous reports.^[1–11,13,17,18]^ In 1994, de Bruin et al observed that EBV positive tumor cells were found in nasal T-cell lymphoma more frequently than in lymphomas originating from other sites.^[19]^ Consequently, in 1997, Petrella et al further described that the EBV expression in NK/T-cell lymphoma shows a site-dependent relationship.^[16]^ More than 90% of patients with nasal NK/T-cell lymphoma involving the nasal cavity and paranasal sinuses consistently showed EBV genomes that seemed to proliferate in clonal and episomal form in the neoplastic cells.^[20]^ There is a strong correlation between the EBV and the CD56 + NK/T-cell lymphomas based on cases reported in the literature. EBV+/CD56– tumors have the same poor prognosis as EBV+/CD56+ tumors, whereas EBV–/CD56+ lesions are somewhat less aggressive.^[10]^ Therefore, the presence of EBV seems to be associated with a worse prognosis and negatively affects patient survival.^[20]^

Different from other types of lymphomas, the treatment for primary testicular NK/T-cell lymphoma is not well established, but radical orchiectomy is the commonest initial therapy, with radiation or chemotherapy used as an adjuvant treatment modality in select cases. Unfortunately, none of the patients were cured of their disease and the mean time to relapse was <6 months in the cases published. Herein, we present 2 nonexclusive hypotheses that may explain the rapid progression of these diseases. The first possibility is an association with EBV infection, increasing the invasive potential of the disease.^[20]^ The second possible contributing factor is the development of multidrug resistance (MDR-1) genes.^[21]^ The presence of MDR-1 genes is also an additional reason for the rapid widespread dissemination of this disease.^[21]^ The highly aggressive course and dismal prognosis have led some investigators to recommend bone marrow or peripheral stem cell transplantation and consolidation chemotherapy in an effort to combat this fatal condition.^[22,23]^

## 4 Conclusions

In conclusion, primary testicular NK/T-cell lymphoma is an uncommon and extremely aggressive malignant tumor presenting unique morphological features and aberrant expression of CD56 as well as EBV. All known patients showed relapse soon after diagnosis despite aggressive treatment, indicative of an extremely poor prognosis. We present this case with detailed clinical and pathological features to inform and educate physicians on the appropriate diagnostic approaches to help guide their investigations and to promote the development of more effective novel treatment strategies to improve the survival rate for patients afflicted by primary testicular NK/T-cell lymphoma.

## 5 Author contributions

**Data curation:** W-L. Zhang.

**Formal analysis:** W-L. Zhang, R. Jug, E. Wang, H-Y. Zhao, H-T. Xu.

**Writing -- original draft:** W-L. Zhang.

**Investigation:** S. Ma, R. Jug, F. Li, L. Cai, C-Q. Yu, S. Shen.

**Supervision:** E. Wang, H-T. Xu, L-H. Yang.

**Validation:** E. Wang.

**Funding acquisition:** H-Y. Zhao, H-T. Xu, L-H. Yang.

**Project administration:** L-H. Yang.

**Resources:** L-H. Yang.

**Validation:** L-H. Yang.

**Writing – review & editing:** L-H. Yang.
